# Development and Biocompatibility Evaluation of Photocatalytic TiO_2_/Reduced Graphene Oxide-Based Nanoparticles Designed for Self-Cleaning Purposes

**DOI:** 10.3390/nano7090279

**Published:** 2017-09-19

**Authors:** Ionela Cristina Nica, Miruna S. Stan, Marcela Popa, Mariana Carmen Chifiriuc, Gratiela G. Pircalabioru, Veronica Lazar, Iuliana Dumitrescu, Lucian Diamandescu, Marcel Feder, Mihaela Baibarac, Marin Cernea, Valentin Adrian Maraloiu, Traian Popescu, Anca Dinischiotu

**Affiliations:** 1 Department of Biochemistry and Molecular Biology, Faculty of Biology, University of Bucharest, 91-95 Splaiul Independentei, 050095 Bucharest, Romania; cristina.nica@bio.unibuc.ro (I.C.N.); anca.dinischiotu@bio.unibuc.ro (A.D.); 2 Department of Botanic-Microbiology, Faculty of Biology, University of Bucharest, 1-3 Aleea Portocalelor, 060101 Bucharest, Romania; marcela.popa@bio.unibuc.ro (M.P.); carmen.chifiriuc@bio.unibuc.ro (M.C.C.); gratiela.pircalabioru@bio.unibuc.ro (G.G.P.); veronica.lazar@bio.unibuc.ro (V.L.); 3 Research Institute of the University of Bucharest–ICUB, University of Bucharest, 91-95 Splaiul Independentei, 050095 Bucharest, Romania; 4 National R&D Institute for Textiles and Leather Bucharest (INCDTP), 16 Lucretiu Patrascanu, 030508 Bucharest, Romania; iuliana.dumitrescu@certex.ro; 5 National Institute of Materials Physics (NIMP), Atomistilor 405A, 077125 Bucharest-Magurele, Romania; mfeder@infim.ro (M.F.); barac@infim.ro (M.B.); mcernea@infim.ro (M.C.); maraloiu@infim.ro (V.A.M.); tr.popescu@gmail.com (T.P.)

**Keywords:** titanium dioxide, photocatalysts, reduced graphene oxide, oxidative stress, antimicrobial, self-cleaning

## Abstract

Graphene is widely used in nanotechnologies to amplify the photocatalytic activity of TiO_2_, but the development of TiO_2_/graphene composites imposes the assessment of their risk to human and environmental health. Therefore, reduced graphene oxide was decorated with two types of TiO_2_ particles co-doped with 1% iron and nitrogen, one of them being obtained by a simultaneous precipitation of Ti^3+^ and Fe^3+^ ions to achieve their uniform distribution, and the other one after a sequential precipitation of these two cations for a higher concentration of iron on the surface. Physico-chemical characterization, photocatalytic efficiency evaluation, antimicrobial analysis and biocompatibility assessment were performed for these TiO_2_-based composites. The best photocatalytic efficiency was found for the sample with iron atoms localized at the sample surface. A very good anti-inhibitory activity was obtained for both samples against biofilms of Gram-positive and Gram-negative strains. Exposure of human skin and lung fibroblasts to photocatalysts did not significantly affect cell viability, but analysis of oxidative stress showed increased levels of carbonyl groups and advanced oxidation protein products for both cell lines after 48 h of incubation. Our findings are of major importance by providing useful knowledge for future photocatalytic self-cleaning and biomedical applications of graphene-based materials.

## 1. Introduction

Right after its first isolation in 2004 [1], graphene (GR) caught scientists’ attention due to its unique physicochemical properties of exceptional thermal and electronic conductivity [2,3], mechanical strength [4], high surface area and facile biological/chemical functionalization [5]. Since then, graphene and its derivatives have been intensively studied for a broad spectrum of applications for GR-based photonics and optoelectronics [6], membrane technology [7], new composite materials [8], chemical and biosensors [9], fuel cells [10], energy storage and conversion [11,12], and electrocatalysis [13,14]. More recently, graphene sheets (GS) and graphene oxide (GO) have proved effective for bacterial inhibition and developed a great potential in photothermal therapy and drug delivery [15].

On the other hand, titanium dioxide (TiO_2_) at nano-level presents high strength and good corrosion resistance [16], as well as photocatalytic activity [17]. Besides its strong oxidative, antibacterial [18], anticorrosive [19] and self-cleaning [20] properties, TiO_2_ has excellent photostability, being also the most available and cheapest photocatalyst currently known [21]. However, the photocatalytic oxidation of organic compounds mediated by TiO_2_ only appears under ultraviolet (UV) radiation, which is one of the major limitations for its use [22]. By combining the semiconductor with some electron acceptors, the electron hole recombination of TiO_2_ under illumination could be prevented [23]. GR can facilitate charge separation and can function as an electron carrier [24]. In consequence, it is a good candidate to form composites with TiO_2_ with enhanced catalytic performances [25]. Several researches on the enhancements in the photocatalytic and antibacterial activity of TiO_2_/GO composites after solar light exposure were published [26,27,28,29,30]. In addition, by doping the TiO_2_ nanoparticles (NPs) with metallic (Fe) and non-metallic (N) atoms, their photocatalytic performance was also expanded in visible light [31].

Research on GR and GO revealed that they maintain stable under physiological conditions and proved excellent biocompatibility with various cell types [32]. For example, it was reported that GO inhibits bacterial growth, inducing a slight toxicity to human alveolar epithelial cells [33]. Also, Bendali et al. showed that neuronal cells can survive on peptide-free graphene layers [34]. Another more recent study claimed that the main mechanism of cytotoxicity of GO is the generation of reactive oxygen species (ROS) [35], which is also one of the most important parameters for photocatalysis. Despite this, there are only few studies regarding the cytotoxicity of GO or reduced graphene oxide (RGO)-based materials on bacterial or mammalian cells.

Therefore, our research addresses a hot topic, which engages the European Community through a dedicated work package of the Graphene Flagship project. Furthermore, to highlight the importance of these layered materials, it should be mentioned that in 2016 the Nobel Prize for Physics was awarded to GR-related materials bismuth chalcogenides [36]. The key novelty of our present work compared to previous studies is represent by the fact that, for this time, RGO were decorated with two sorts of TiO_2_ NPs co-doped with 1% iron and nitrogen atoms (TiO_2_-Fe(1%)-N NPs) and synthesized under hydrothermal conditions as follows: one of them (sample A) was obtained by a simultaneous precipitation of Ti^3+^ and Fe^3+^ ions to achieve their uniform distribution, and the other one (sample B) after a sequential precipitation of these 2 cations for obtaining a higher concentration of iron on the surface of Ti^4+^ oxohydroxide. In terms of structure and morphology, these nanocomposites were investigated by X-ray diffraction (XRD), Mössbauer and Raman spectroscopy, and also by transmission electron microscopy (TEM). The photocatalytic activity of these powders was comparatively tested on the methylene blue (MB) degradation and revealed by a photocatalytic checker. In addition, the antimicrobial efficiency of the new-developed TiO_2_/RGO nanocomposites and their biological effects on human cells were analyzed. The antimicrobial activity against reference bacterial strains including Gram negative *Escherichia coli* and *Pseudomonas aeruginosa* as well as Gram-positive *Staphylococcus aureus* was tested using quantitative assays. Further, the cytotoxic effects were determined in vitro on normal human dermal (CCD 1070Sk cell line) and pulmonary (MRC-5 cell line) fibroblasts by measuring several parameters, such as cell viability and morphology, protein oxidation and lipid peroxidation, reduced glutathione levels and nitric oxide level as well as lysosomes accumulation.

## 2. Results

### 2.1. Physico-Chemical Characteristics of TiO_2_/Reduced Graphene Oxide Nanocomposites

#### 2.1.1. XRD Analysis

**Figure 1:**
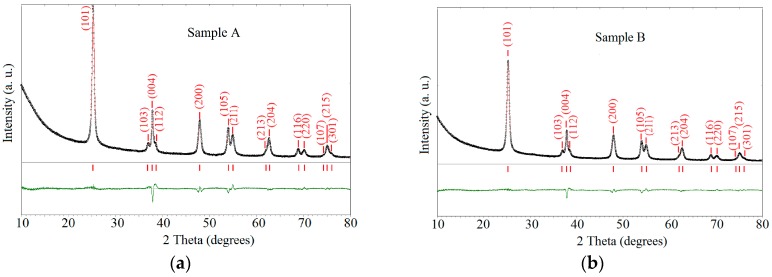
XRD patterns of the two TiO_2_/RGO nanocomposites samples: calculated curve in red color; difference in green color.

XRD patterns of the samples A and B are depicted in Figure 1 together with the Rietveld refinement analysis. Rietveld refinement data (as lattice parameters c and a, cell volume V and Ti and O coordinates) shown in Table 1 in comparison with bulk anatase prove the unique presence of anatase phase in both samples. From Scherer equation [37] the mean crystalline size (Φ) of the anatase nanoparticles is close to 18 nm. The lattice parameters, the microstrain (ε) and the Ti and O atom position indicate the structural similarity of the two samples.

#### 2.1.2. Mössbauer Spectroscopy

**Figure 2:**
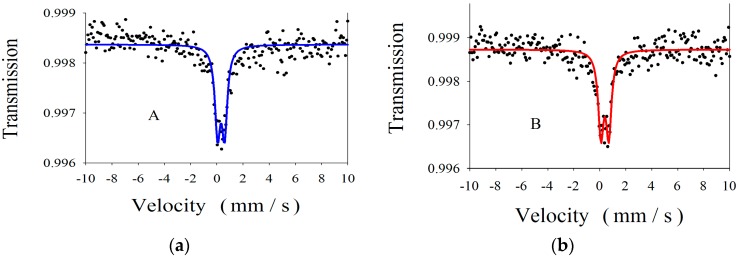
Room temperature Mössbauer spectra of the nanocomposites samples A (**a**) and B (**b**), and the computer fit (continuous lines).

In order to find out the valence state and the localization of iron ions in our samples, room temperature recorded ^57^Fe Mössbauer spectra were analyzed for both samples A and B. Figure 2 displays both spectra and the computer fit (continuous red and blue lines). The spectra consist in a central quadrupole pattern. In the hypothesis of Lorentzian line, the computer fit reveals the presence of a single quadrupole doublet for each sample. In Table 2 the obtained hyperfine parameters, isomer shift (IS) and quadrupole splitting (ΔE_Q_) are characteristic for Fe^3+^ ions in octahedral symmetry [38]. The higher IS value in the case of sample B with respect to sample A indicates a decrease of electronic charge density at Fe nucleus. The quadrupole splitting is also higher in the sample B. To explain these findings we need to remember that at nanometer scale a large fraction of atoms can be found at the particle surface. The atom coordination is drastically distorted leading to large ligand-field splitting. In the case of iron (Mössbauer element in our samples) this distortion is reflected in the ΔE_Q_ and isomer shift IS values. ΔE_Q_ is a measure of the hyperfine interaction between the electric field gradient (a prevailing component coming from the lattice contribution) and a nuclear quadrupole moment. A more distorted lattice leads to a higher quadrupole splitting. The electronic charge density at the Mössbauer nucleus is also very sensitive to the lattice distortion, higher IS reflecting a decrease of electronic charge at Mössbauer nucleus [38]. The superior ΔE_Q_ and IS values found in the case of sample B in comparison with sample A support the hypothesis of more iron atoms localized at the particle surface in sample B. This effect could be considered as a result of sequential precipitation of Ti^3+^ followed by Fe^3+^ in the case of sample B.

#### 2.1.3. Raman Spectroscopy

In order to understand the Raman spectroscoy results, we have to remember that in the synthesis we used pristine grafene oxide (GO), and poly diallyl dimethyl ammonium chloride (PDDA) as a reducing agent (see Section 4.2).

**Figure 3:**
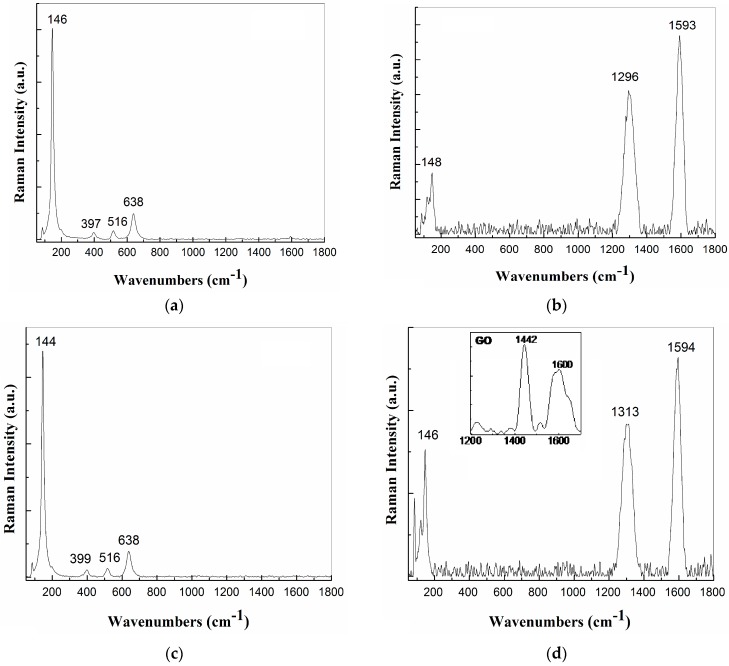
Raman spectra recorded at excitation wavelength of 1064 nm for: sample A without GO (**a**), sample A synthesized in the presence of GO (**b**), sample B without GO (**c**) and sample B synthesized in the presence of GO dispersed in H_2_O. The insert in Figure 3**d** shows the Raman spectrum of pristine GO dispersed in H_2_O.

Figure 3 displays the Raman spectra of TiO_2_-Fe(1%)-N samples obtained with simultaneous co-precipitation (Figure 3a) and sequential co-precipitation (Figure 3c) as well as the two samples of TiO_2_-Fe(1%)-N decorated GO (Figure 3b,d). At present, it is known that anatase has a tetragonal structure with spatial group D_4h_ (*I*4_1_/amd), this having six vibrational modes active in Raman spectroscopy A_1g_, 2B_1g_ and 3E_g_ [39]. According to Figure 3a,c, the five Raman lines peaked at 144–146, 197, 397–399, 516 and 638 cm^−1^ are assigned to the vibrational modes E_g_, E_g_, B_1g_, A_1g_ + B_1g_ and E_g_, respectively; they belong to TiO_2_ phase with anatase structure [40]. The main peak at ~144–146 cm^−1^ coming from external vibrations of anatase structure is well defined, indicating the crystallization of anatase phase in the two samples. The difference in the position of the Eg vibrational mode, accompanied of a variation in full width at half-maximum of the main anatase, was interpreted by S. K. Gupta et al. [41] to be an evidence of the variation in the size of the TiO_2_ NPs. In our case, the difference in the position of the anatase Eg vibrational mode is not accompanied of a variation in full width at half-maximum of the main anatase mode. Therefore, we are tempted to assign the change in the position of the Eg vibrational mode to a doping process. X-ray photoelectron spectroscopy (XPS) was used to reveal the presence of nitrogen in the samples, as in our previous study [42]. The total nitrogen content detected by XPS, with respect to the total TiO_2_ was ~0.5%. Other Raman lines belonging to rutile TiO_2_ phase (235 cm^−1^, 447 cm^−1^ and 612 cm^−1^) were not identified.

**Figure 4:**
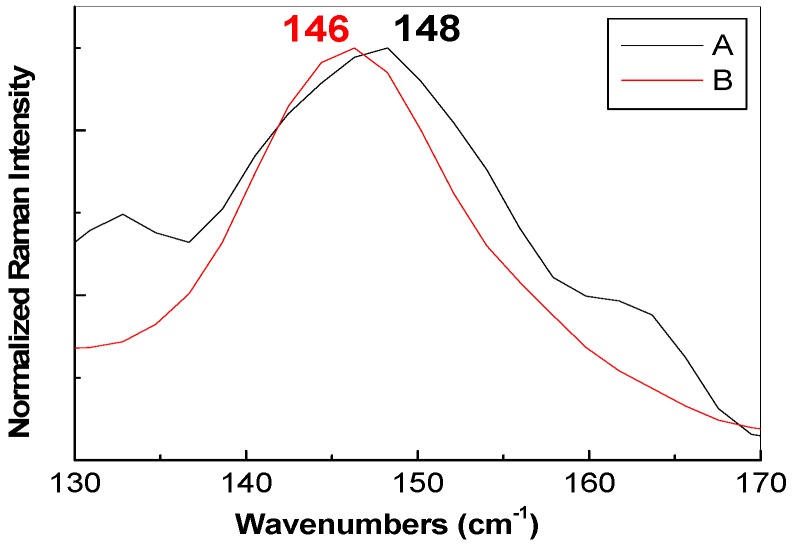
The spectral range 130–170 cm^−1^ in the case of Raman spectra recorded at excitation wavelength of 1064 nm for: sample A synthesized in the presence of GO (black curve), and sample B synthesized in the presence of GO dispersed in H_2_O (red curve).

As observed in the insert of Figure 3d, the main Raman lines of the pristine material GO sample dispersed in H_2_O are peaked at 1442 and 1600 cm^−1^, they being often labeled as D and G bands, which were assigned to the breathing vibration mode of the carbon hexagonal rings and the E_2g_ phonon mode at the center of the Brillouin zone [43]. The position of the D and G Raman bands of GO dispersed in H_2_O is significant shifted in comparison with the samples prepared according to previous studies [44,45], when Raman spectra, obtained under the excitation wavelength of 1064 nm, were characterized by the D and G bands situated at: (i) 1350 and 1600 cm^−1^ in the case of GO; and (ii) at 1294 and 1606 cm^−1^ for RGO. A brief analysis of Figure 3b,c indicates that the Raman spectra of the GO decorated with TiO_2_-based NPs show: (i) the D and G Raman bands down-shifted from 1442 and 1600 cm^−1^ to ~1294–1311 and 1593–1594 cm^−1^, respectively (a similar behavior of the G band was reported for graphene-TiO_2_ composites synthesized by L.M. Pastrana-Martinez et al. [46]; this down-shift of the G band indicates that a reduction of GO took place); and (ii) an up-shift of the E_g_ mode of TiO_2_ from 146 cm^−1^ (Figure 3a) and 144 cm^−1^ (Figure 3c) to 148 cm^−1^ (Figure 3b) and 146 cm^−1^ (Figure 3d), respectively. The upshift of the Eg mode of the anatase TiO_2_ samples synthesized in the presence of GO indicates an increase in the size of TiO_2_ NPs. This sentence is supported by (i) the position of the Eg vibrational mode, accompanied of a variation in full width at half-maximum of the main anatase as shown in Figure 4 and (ii) above XRD analysis. Figure 3b,d also highlight a change of the ratio between the relative intensities of D and G bands (I_D_/I_G_) from 1.4 (insert of Figure 3d) to 0.75 (Figure 3b) and 0.69 (Figure 3d), respectively, as a result of the decoration of the RGO sheets with TiO_2_-Fe(1%)-N NPs when a variation of the defects in the graphene basal plane took place.

#### 2.1.4. TEM Characterization

**Figure 5:**
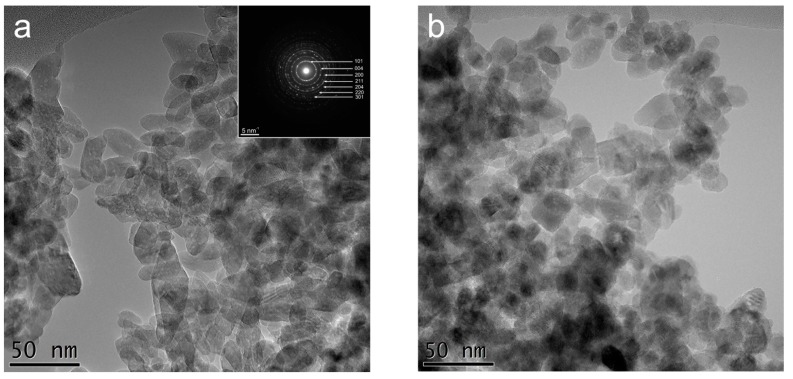

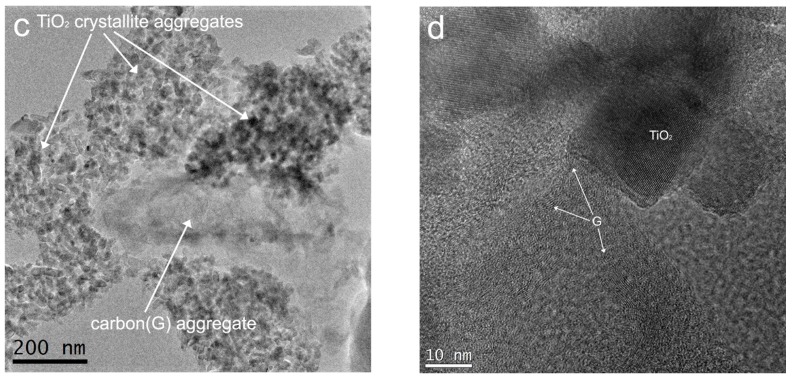
(**a**) CTEM images of TiO_2_ nanoparticles for sample A and SAED pattern (inset); (**b**) CTEM images of TiO_2_ nanoparticles for sample B; (**c**) CTEM images of TiO_2_ nanoparticles and carbon (G) aggregate in sample B; (**d**) HRTEM image of TiO_2_ nanoparticles and carbon (G) layers around.

Conventional TEM (CTEM) images obtained for the samples A and B (Figure 5a,b) revealed that TiO_2_ nanoparticles have different morphologies (spherical, cubic, ellipsoidal) and various sizes. Graphene aggregates can be also observed in Figure 5c. Selected Area Electron Diffraction (SAED) patterns (Figure 5a—inset) demonstrated that TiO_2_ NPs are crystallized in anatase structure with the space group *I*4_1_/amd. High resolution TEM (HRTEM) image (Figure 5d) of sample B showed a crystallized TiO_2_ NP with graphetized carbon at the surface.

**Figure 6:**
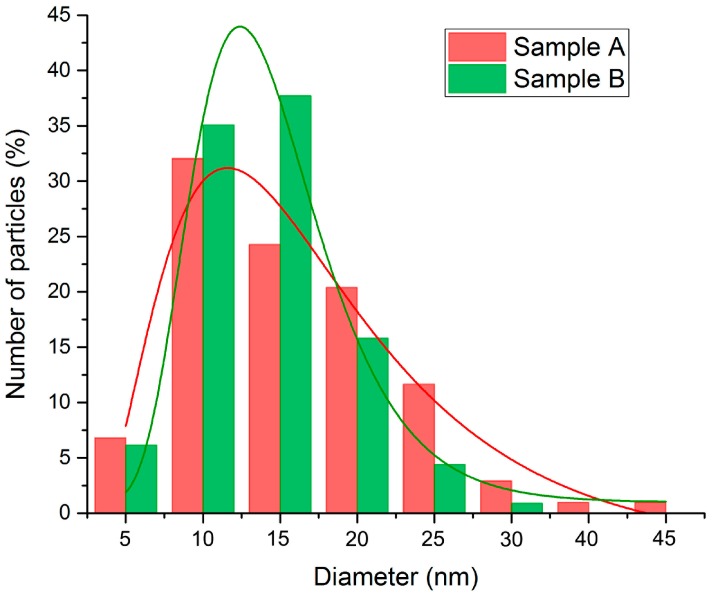
Size distributions of TiO_2_ nanoparticles for samples A and B and the corresponding LogNormal fittings.

CTEM images showed that the size of TiO_2_ NPs belongs to the interval of 7 to 44 nm for sample A and 8 to 35 nm for sample B (Figure 6), the mean particle size being 17.8 nm and 15.8 nm, respectively. The particle size distribution is represented by a Log Normal distribution function (Figure 6). These results are in accordance with the XRD measurements revealed in Figure 1.

#### 2.1.5. Photocatalytic Activity

**Figure 7:**
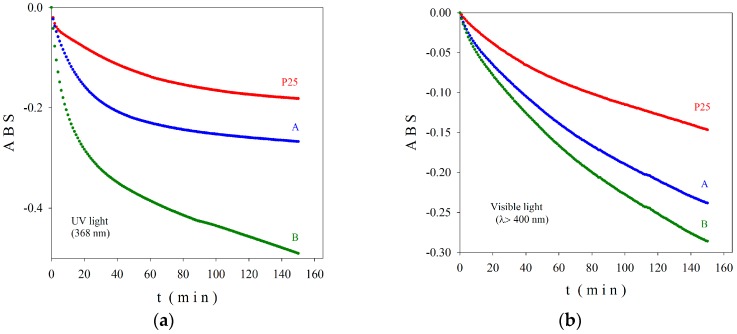
Absorbance (ABS) in UV (**a**) and visible (**b**) light for the investigated samples A and B, in comparison with the commercial product P25 Degussa.

The absorbance (ABS) values of samples A and B, in comparison with the commercial product Degussa P25, are depicted in Figure 7. As described in Section 4.3. Characterization of Photocatalysts of this article, ABS is a measure of the photocatalytic efficiency of sample, higher negative values indicating better activity [47]. One can observe that in 150 min one half of MB amount was decomposed by the photocatalytic substrate (sample B) under UV irradiation and nearly 30% under visible light exposure. The sample B, where the Fe^3+^ ions are located at the surface, exhibited superior activity in both UV and visible light region. Both samples A and B presented better photocatalytic activity compared to P25 particles. After 150 min of irradiation, an exponential decay behavior and no saturation effects can be claimed for the photocatalytic degradation of the investigated samples especially in visible light.

To explain the results in Figure 7, we have to remember that a photocatalytic reaction takes place at the surface on a semiconductor due to the reactive species generated by electron-holes pairs under irradiation with light having appropriate energy (greater than the band gap energy). The literature on photocatalysis reveals the correlation between structure, morphology, cation or anion doping level, pollutant nature and concentration, and the photocatalytic properties of semiconducting materials. The commercial product Degussa P25 consists of two TiO_2_ phases: ~85% anatase and ~15% rutile. Samples A and B consist only in nanoscaled anatase phase co-doped with iron and nitrogen decorating RGO. Moreover, due to a sequential precipitation Ti^3+^ and Fe^3+^ in the preparation of sample B, the iron ions are placed at the sample’s surface as evidenced by Mössbauer spectroscopy. It is believed that the synergetic effects of Fe, N doping of TiO_2_ NPs decorating RGO consist mainly in the increase of the electron-hole life time, involving an increase of photocatalytic effects, as evidenced also in our measurements (Figure 6).

### 2.2. Antimicrobial Properties of TiO_2_/Reduced Graphene Oxide Nanocomposites with Planktonic and Biofilm Embedded Microbial Cells

**Figure 8:**
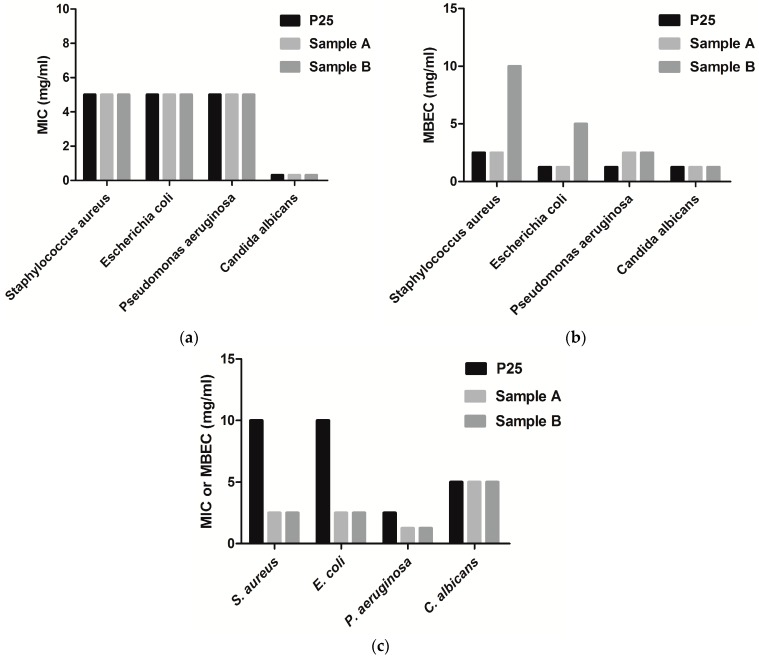
MIC and MBEC values of the investigated samples A and B in dark (**a**,**b**) and under light exposure (**c**), in comparison with the commercial product P25 Degussa.

The antimicrobial efficiency was measured in this study in dark conditions (Figure 8a,b) and under light exposure (Figure 8c) in order to provide a comparative analysis. The two tested samples of TiO_2_-RGO nanocomposites exhibited a very good antimicrobial activity under light exposure against both Gram-positive and Gram-negative tested strains grown in planktonic state compared to results obtained in dark, with minimum inhibitory concentration (MIC) values four times lower than that of commericial P25 Degussa particles in case of *S. aureus* and *E. coli* strains, and two times lower in case of *P. aeruginosa* strains (Figure 8c). In contrast, the antifungal activity of the photocatalysts was similar to that of P25 particles, for both types of exposure (Figure 8). This lower efficiency on *C. albicans* compared to the antibacterial effect was most probably due to the complex cell wall structure and different metabolism of fungi. Same observations were also noted previously by another group of researchers on graphene oxide [48].

A similar activity against planktonic microorganisms (Figure 8a) and, but a different anti-biofilm efficiency between the two nanocomposites were recorded in dark (Figure 8b). In exchange, no differences in the intensity and spectrum of the antimicrobial activity of the two samples obtained by simultaneous co-precipitation or by sequential precipitation were observed against the biofilm and planktonic cells when light was used (Figure 8c), as revealed by the similar MIC and minimum biofilm eradication concentration (MBEC) values, suggesting that iron distribution did not influence the photocatalytic antimicrobial efficiency.

### 2.3. Effects of TiO_2_/Reduced Graphene Oxide Nanocomposites on Skin and Lung Fibroblasts

#### 2.3.1. Cell Viability

**Figure 9:**
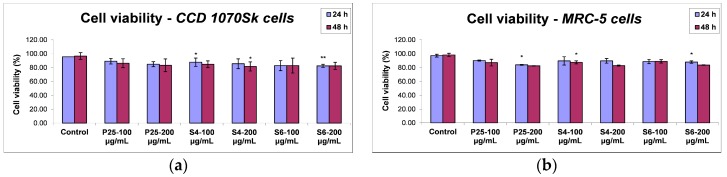
Biocompatibility of two TiO_2_/RGO nanocomposites samples (A and B) and commercially available TiO_2_ NPs (P25), as shown by cell viability assay at 24 and 48 h exposure for normal skin (**a**,**c**) and lung (**b**,**d**) cells. Data are calculated as means ± standard deviation (SD) (*n* = 3) and showed relative to control (untreated cells). * *p* < 0.05 and ** *p* < 0.01 vs. control.

**Figure 10:**
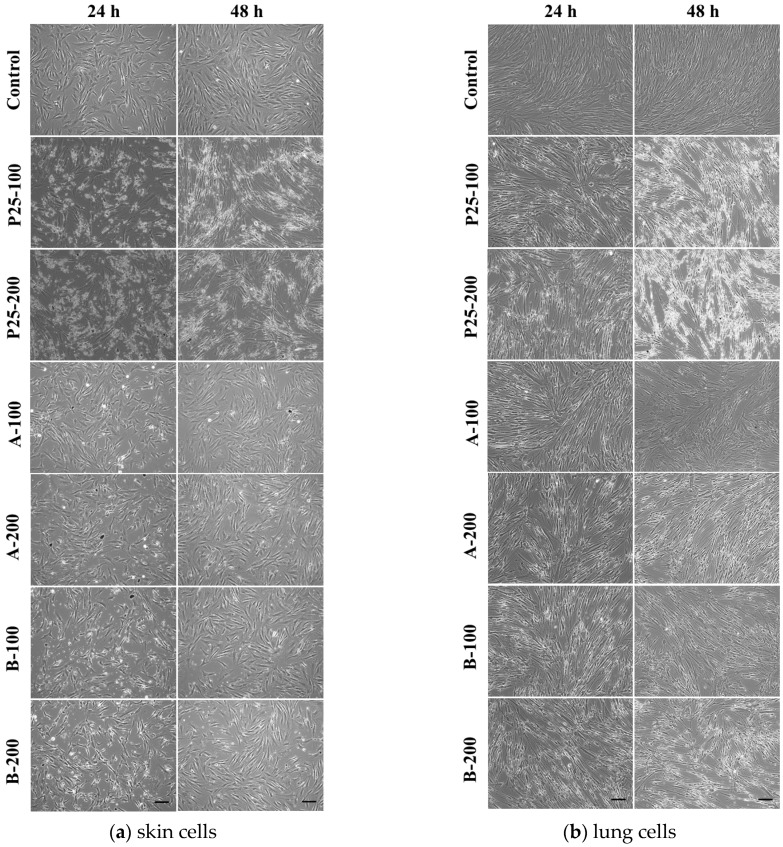
Representative images of phase contrast microscopy revealing cellular morphology of CCD 1070Sk (**a**) and MRC-5 (**b**) fibroblasts exposed to 100 and 200 μg/mL of two TiO_2_/RGO nanocomposites samples (A and B) and commercially available TiO_2_ NPs (P25) for 24 and 48 h. Scale bar: 100 μm.

The influence of TiO_2_/RGO composites on the cell viability and their potential to activate inflammation were assessed by in vitro methods performed on normal human skin and lung fibroblast cells. Two different intervals of incubation (24 and 48 h) and two doses (100 and 200 μg/mL) were chosen for each of the three photocatalytic samples: graphene oxide decorated with two different types of TiO_2_-Fe(1%)-N NPs and commercial TiO_2_ P25 Degussa. Firstly, the toxic effects of graphene-based nanocomposites exposure on cell viability were measured by Trypan Blue assay. Figure 9a,b revealed that, regardless of the exposure time or photocatalyst dose tested, both lung and dermal cells did not show any significant changes regarding the cell viability. The average percentages of cell viability for control after 24 and 48 h were 96.55% ± 2.01 and 97.25% ± 4.87, respectively. These data are calculated as the mean ± standard deviation (SD) of three independent measurements, and three count repetitions were performed for each batch of cells. Following incubation with the photocatalytic samples, the cell viability was not less than 80%. These findings were in agreement with the phase contrast microscopy observations (Figure 10).

**Figure 11:**
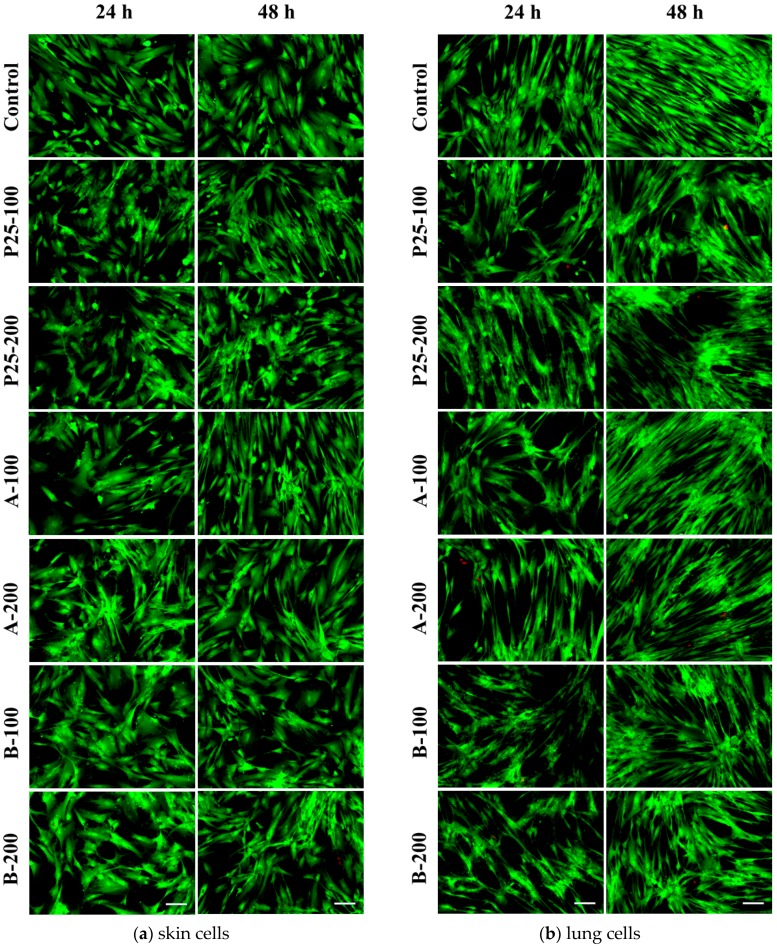
Viability of skin (**a**) and lung (**b**) cells at 24 and 48 h exposure to 100 and 200 μg/mL of the two TiO_2_/RGO nanocomposites samples (A and B) and commercially available TiO_2_ NPs (P25). Live/Dead assay was used to stain the viable cells (green) and dead cells (red). Scale bar: 100 μm.

To better describe the cytotoxicity of TiO_2_/RGO composites, fluorescence microscopy investigations were done using a mix of dyes: calcein AM and ethidium bromide. Consequently, live cells developed a green fluorescence after the ester bond hydrolysis of calcein AM to calcein, while dead cells appeared red based on the ethidium homodimer binding to DNA in cells with disrupted nuclear membranes. Thus, the microscopic observations shown in Figure 11 within this test were in concordance with the results of Trypan Blue assay, revealing similar cell viabilities for the analyzed samples.

#### 2.3.2. Inflammatory Response

**Figure 12:**
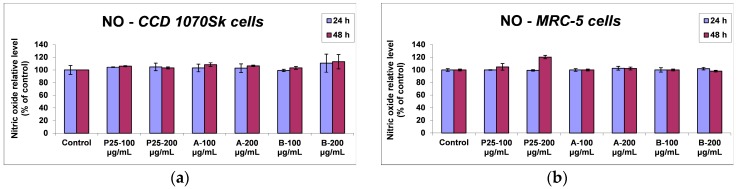
Inflammatory response induced in normal skin (**a**) and lung (**b**) cells at 24 and 48 h exposure to 100 and 200 μg/mL of the two TiO_2_/RGO nanocomposites samples (A and B) and commercially available TiO_2_ NPs (P25), as shown by nitric oxide (NO) released in the media. Data are calculated as means ± standard deviation (SD) (*n* = 3) and showed relative to control (untreated cells).

The amount of released nitric oxide (NO) postexposure by the two types of cells was also measured. No considerable differences were evidenced between the two cell types or the three samples. Therefore, the value of NO released by CCD 1070Sk cells in the media was maintained near the control level regardless the photocatalyst dose or time of incubation, as shown in Figure 12a, while MRC-5 lung cells registered a slight increase of NO concentration, up to 20% of control after 48 h of incubation with 200 μg/mL P25 Degussa particles (Figure 12b). Thus, the level of inflammation occurred consequently to the exposure to tested nanocomposites was very low and it cannot be appreciated as significant.

#### 2.3.3. ROS Generation and Cellular Antioxidant Defense

**Figure 13:**
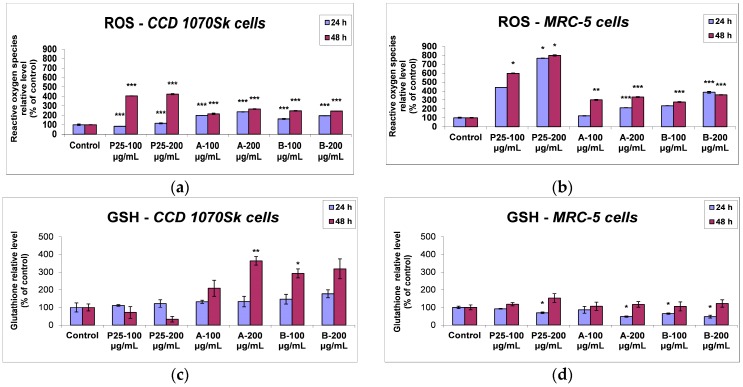
ROS and GSH levels in normal skin (**a**,**c**) and lung (**b**,**d**) cells at 24 and 48 h exposure to 100 and 200 μg/mL of the two TiO_2_/RGO nanocomposites samples (A and B) and commercially available TiO_2_ NPs (P25). Data are calculated as means ± standard deviation (SD) (*n* = 3) and showed relative to control (untreated cells). * *p* < 0.05, ** *p* < 0.01 and *** *p* < 0.001 vs. control.

To assess if TiO_2_/RGO composites induced oxidative stress, the level of intracellular ROS was measured using the fluorescence intensity of dichlorofluorescein (DCF). The exposure of skin cells to the nanocomposites generated an increase in ROS level, as their production happened quickly, from the first 24 h of interaction between cells and nanophotocatalysts (Figure 13a). The incubation with P25 particles did not significantly change the ROS level in the first 24 h, but after 48 h the radical generation rate was 4.0 times (for 100 μg/mL) and 4.2 times (for 200 μg/mL) higher in comparison with control cells, showing a significant potential of toxicity. In contrast, after 24 h of treatment with the RGO-based nanocomposites, the levels of ROS were 1.6 and 1.9-fold higher than control dermal cells for 100 μg/mL concentration of samples A and B, respectively, reaching up to 2.4-fold higher for the 200 μg/mL dose of sample A. The fluorescence intensity of DCF elevated during the nanocomposites incubation in a concentration-dependent manner, with no major differences between the two samples and the two time intervals. TiO_2_/RGO nanocomposites had a more pronounced effect on MRC-5 cells and led to a higher increase of ROS levels (Figure 13b), suggesting that the lung fibroblasts were more susceptible to the nanocomposites exposure than the skin cells. However, a major difference between the P25 NPs and the two photocatalysts containing graphene oxide was observed, the latter ones being less toxic to human cells.

ROS generated as a result of the cells normal metabolism as well as due to xenobiotic exposure can be counteracted by non-enzymatic and enzymatic antioxidant systems.

In order to analyse the non-enzymatic antioxidant defense system responsible for the cell protection against oxidative injuries induced by ROS, the reduced glutathione (GSH) levels were assessed. Thus, Figure 13c,d shows an enhancement of GSH content both in the dermal and lung fibroblasts exposed to 200 μg/mL of samples A and B for 48 h, up to 3.6 and 1.5 times of control, respectively, proving the activation of GSH-based antioxidant mechanisms for an adequate cellular defense against oxidative injuries. Taking into account that GSH can directly interact with ROS, quenching them, the strong diminution of the GSH level in skin cells by almost 70% after 48 h exposure to P25 particles correlates with the high level of ROS. Also, the lower level of ROS noticed for both doses of A and B nanocomposites compared to P25 NPs could be correlated with the raised concentration of GSH induced by them (Figure 13c).

In contrast, not only P25 NPs, but also TiO_2_/RGO composites excited a more significant influence on MRC-5 cells, as it was revealed by the dose-dependent decrease of the GSH content after 24 h of incubation, followed by an increase after 48 h up to control level or higher.

#### 2.3.4. Lipid Peroxidation

**Figure 14:**
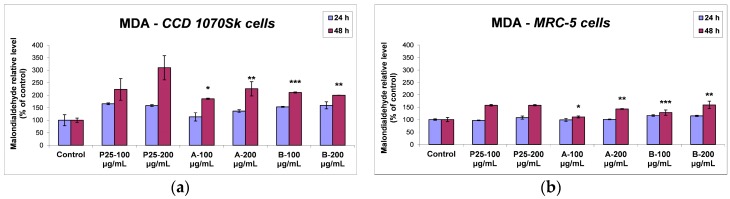
MDA levels measured for normal skin (**a**) and lung (**b**) cells exposed at 24 and 48 h exposure to 100 and 200 μg/mL of the two TiO_2_/RGO nanocomposites samples (A and B) and commercially available TiO_2_ NPs (P25). Data are calculated as means ± standard deviation (SD) (*n* = 3) and showed relative to control (untreated cells). * *p* < 0.05, ** *p* < 0.01 and *** *p* < 0.001 vs. control.

The effects induced on lipid peroxidation by TiO_2_/RGO nanocomposites in normal human dermal and lung fibroblasts are represented in Figure 14. For both types of cells exposed for 24 h to all three kinds of NPs, the malondialdehyde (MDA) content was not significantly changed compared to control. For CCD 1070SK cells, the 48 h incubation with 200 μg/mL of P25 NPs increased the MDA level by almost 200% of control. No considerably distinction was observed between samples A and B, the MDA level increase being of about 100% of control for TiO_2_/RGO samples. Once again, the P25 sample showed a cytotoxic potential higher than graphene oxide-based nanocomposites. The amount of lipid peroxidation in MRC-5 cells was lower in comparison with that one measured in skin cells after the incubation with the three types of particles. The first 24 h in the presence of the nanophotocatalysts did not induce significant changes in the MDA level of MRC-5 cells, but after another 24 h of incubation, the lung cells recorded the same elevation of MDA content, by 57% of control for 100 and 200 μg/mL doses of P25 samples, while the samples A and B determined a dose-dependent MDA accumulation.

#### 2.3.5. Protein Oxidation

**Figure 15:**
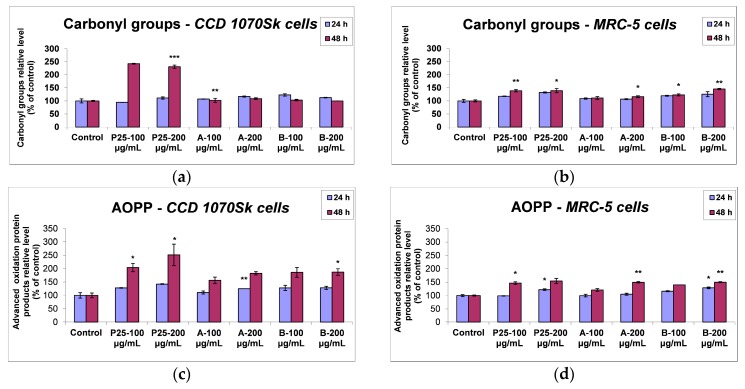
Protein oxidation profile as shown by carbonyl groups (**a**,**b**) and AOPP (**c**,**d**) levels in normal skin and lung cells at 24 and 48 h exposure to 100 and 200 μg/mL of the two TiO_2_/RGO nanocomposites samples (A and B) and commercially available TiO_2_ NPs (P25). Data are calculated as means ± standard deviation (SD) (*n* = 3) and showed relative to control (untreated cells). * *p* < 0.05, ** *p* < 0.01 and *** *p* < 0.001 vs. control.

The level of the protein oxidation was examined to show the possible consequences which can be induced by the elevated ROS level on the redox state of proteins after TiO_2_/RGO nanocomposites exposure. The protein oxidation represented by carbonyl groups, formed at the side chains of cytoplasmic proteins, and by advanced oxidation protein products (AOPP), is shown in Figure 15. In CCD 1070Sk cells (Figure 15a), the incubation with the samples for 24 and 48 h did not significantly change the level of carbonyl groups compared to control, except the commercially P25 NPs which induced an increase of carbonyl groups amount by almost 45% after 48 h of exposure. Instead, the lung fibroblasts registered a different patern of changes in carbonyl groups level (Figure 15b). If the MRC-5 cells treated with the sample A behaved like the control ones, the exposure to 200 μg/mL of sample B led to an increase of carbonyl groups by 45% after 48 h, which was comparable with that induced by P25 NPs.

Moreover, the AOPP levels induced by P25 and nanocomposites exposure in skin and lung cells were assessed. The exposure of CCD 1070Sk cells to samples for 24 h (Figure 15c) induced only a slight increase of the AOPP amount dependent to the particles’ concentration. However, after 48 h in the presence of P25 NPs, the AOPP content registered an increase up to 100% and 150% of control levels for 100 and 200 μg/mL doses, respectively. Instead, samples A and B behaved similarly, inducing an increase in AOPP level by only 86% after 48 h of incubation. The lung fibroblasts registered lower levels of the AOPP content compared to the dermal cells (Figure 15d). Therefore, in the first 24 h of treatment, AOPP level was maintained around control values, except the highest dose (200 μg/mL), which induced a slight increase for all of the three samples that did not exceed 30% of control. After 48 h of incubation with P25 NPs and the two TiO_2_/RGO nanocomposites, the AOPP level continued to elevate in a dose-dependent manner with no major differences between the samples, the highest increase with almost 55% of control being registered in the case of both doses of P25 NPs.

#### 2.3.6. Actin Cytoskeleton Changes

**Figure 16:**
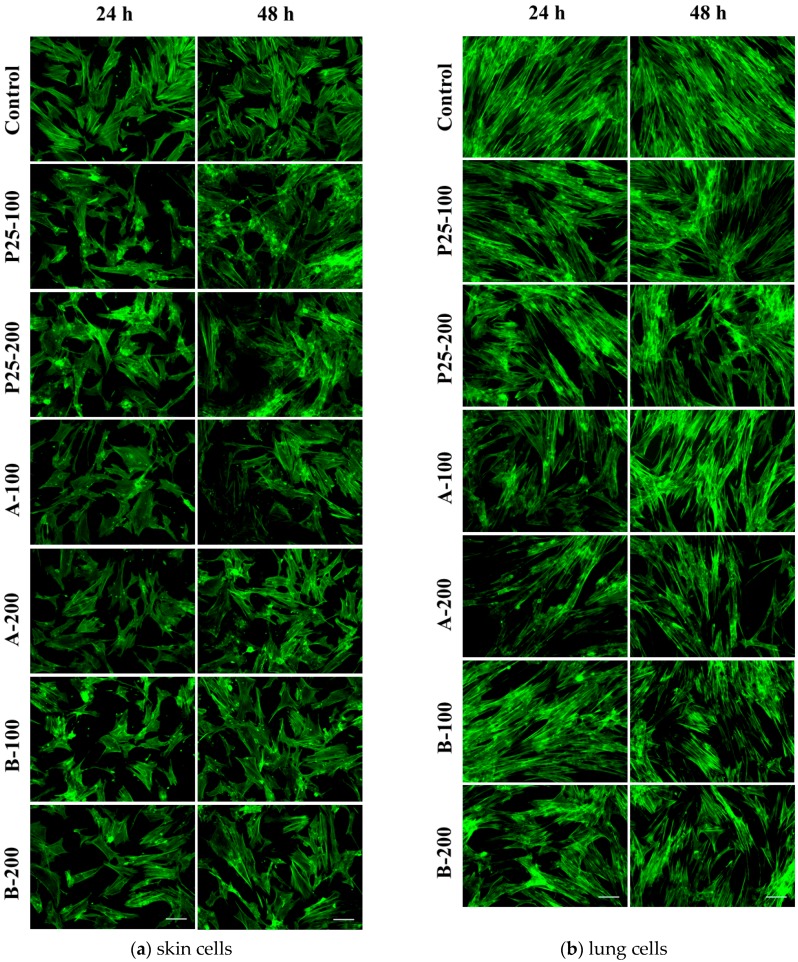
Fluorescence images showing cytoskeletal actin filaments in skin (**a**) and lung (**b**) fibroblasts after 24 and 48 h of exposure to 100 and 200 μg/mL of the two TiO_2_/RGO nanocomposites samples (A and B) and commercially available TiO_2_ NPs (P25). F-actin (green) staining was done by phalloidin-fluorescein isothiocyanate (FITC). Scale bar: 100 μm.

Modifications of CCD 1070Sk and MRC-5 cell morphology following the incubation with TiO_2_/RGO nanocomposites and commercially available TiO_2_ NPs (P25) were investigated by phase contrast and fluorescence microscopy. The organization of actin cytoskeleton displayed in Figure 16 was in accordance with the results of cell viability showed in Figure 9, Figure 10 and Figure 11. Therefore, the normal human skin and lung fibroblasts maintained their elongated morphology, being able to establish multiple focal adhesions at 24 and 48 h of treatment. Also, there were no important changes between the samples in comparison with the control for both cell types. These findings demonstrated that the cell behaviour did not suffer important changes in the presence of nanophotocatalysts.

#### 2.3.7. Lysosomes Distribution and Accumulation

**Figure 17:**
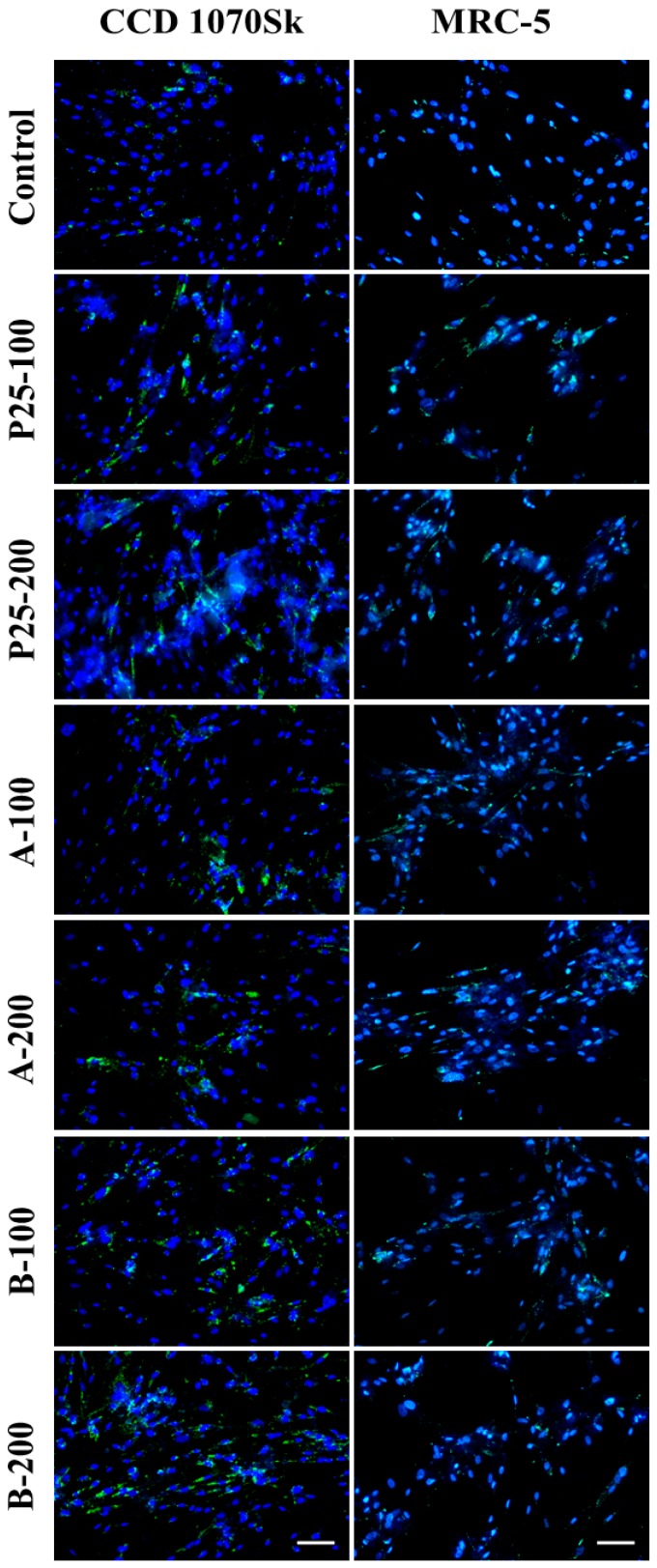
Lysosomes profile in skin (CCD 1070Sk) and lung (MRC-5) fibroblasts after 48 h exposure to different concentrations (100 and 200 μg/mL) of the two TiO_2_/RGO nanocomposites samples (A and B) and commercially available TiO_2_ (P25). Lysosomes (green) staining was done with LysoTracker Green DND-26 and nuclei (blue) were counterstained with Hoechst 33342. Scale bar: 100 μm.

A significant dose-dependent accumulation of lysosomes was revealed in dermal fibroblasts exposed to the all three kinds of photocatalytic samples (Figure 17), these results being consistent with the other tests that underline the induction of oxidative stress in this type of cells after 48 h. Particle sequestration in lysosomes reveals the cell-activated mechanism in order to limit the harmful effects that can be generated by the tested particles. In contrast, a control-like distribution of the lysosomes was noticed for the MRC-5 cells incubated with P25 NPs or TiO_2_/RGO nanocomposites, suggesting a decrease of their toxicity on the lung cells compared to the skin fibroblasts.

## 3. Discussion

The excessive use of antimicrobial agents over years has triggered the selection and dissemination of drug resistant pathogens that are increasingly reported, posing serious risks for patients and raising the necessity of finding novel alternatives for the antimicrobial treatment. One of the most promising approaches is to use engineered nanostructures to overcome microbial drug resistance. Their antimicrobial efficiency can rely on their high surface-to-volume ratio and on their intrinsic or chemically incorporated antibacterial activity [49]. TiO_2_ NPs have a large variety of applications in different fields, including the development of antimicrobial strategies, due to their wide spectrum of microbiocidal and virulicidal effects, occurred as result of photocatalytic reactions [50]. However, during synthesis, similar to other inorganic NPs, TiO_2_ particles tend to agglomerate resulting in the loss of their nanoscale properties. GO represents an oxidized derivative of graphene which, due to its huge surface area and strong hydrophilic character, can easily bind organic and inorganic molecules [51]. Also, the functional groups present on the surface of GO can serve as nucleation and growth sites for NPs [52]. Furthermore, the integration of GO with inorganic NPs permits the adjustment of desired properties for the obtained nanocomposites on order to achieve specific applications. It has been shown that cotton fabrics coated with Fe, N co-doped TiO_2_ NPs showed excellent photocatalytic self-cleaning and antimicrobial properties [53].

The aim of our research was to develop biocompatible TiO_2_/RGO-based nanocomposites with higher photocatalytic activity and antibacterial properties. Therefore, in this paper we describe for the first time the antimicrobial activity of RGO decorated with two types of TiO_2_ particles co-doped with 1% Fe and N. The obtained results showed that the two types of nanocomposites exhibited a very good antibacterial activity, against Gram-positive and Gram-negative strains, with similar intensity against bacteria grown in planktonic and biofilm state under light exposure. These data recommend the obtained nanocomposites for developing efficient anti-biofilm strategies.

Given the potential biomedical applications of TiO_2_/RGO nanocomposites, a systematic evaluation of their biological response initiated after mammalian cell exposure is required. So far, the adverse effects of GO and its derivatives have been deeply analyzed using both in vitro and in vivo assessments. It was shown that GO does not penetrate the membrane cell, but in high concentrations can cause a dose-dependent oxidative stress leading to a slight decrease of cell viability [54]. Also, other research showed that smaller size of particles and higher degree of oxidation could improve biocompatibility of graphene-based nanomaterials [55]. It is already known that TiO_2_ and GO particles are biocompatible with human osteoblasts [56], human dental follicle stem cells [57] and can induce more ROS than single TiO_2_ NPs in aquatic species such as *Daphnia magna* and *Oryzias latipes* [58], but there are still few studies regarding the cytotoxicity of TiO_2_–based nanocomposites. Therefore, our research proposed to expand the limited knowledge available at this moment by evaluating the cytotoxic effects induced by the newly-developed TiO_2_/GO nanocomposites in human lung (MRC-5) and skin (CCD 1070Sk) cells.

In order to describe the adverse effects of nanomaterials, several mechanisms of toxicity have been proposed; these include the induction of oxidative stress that has been discussed by many researchers. It is widely accepted that high levels of ROS trigger lipid peroxidation in the cell, mitochondrial and nuclear membranes, which further induces degradation of cytosolic proteins and DNA damages [59]. Our experiments highlighted a dose- and time-dependent elevation of ROS levels, suggesting the potential of these nanocomposites to induce the formation of ROS as a result of the photocatalytic process [60].

There are several studies regarding the influence of visible light exposure on cell cultures. Components of the visible spectrum can cause cellular dysfunction and even death. The main targets of light exposure are mitochondria, which are laden with chromophores (cytochrome C oxidase, flavins and flavoproteins) and can release several ROS, including singlet oxygen, superoxide, and hydroxyl radicals [61]. Also, it was reported that irradiation of skin cells with visible light can induce significant ROS production, which mediates the release of proinflammatory cytokines and matrix-degrading enzymes expression [62]. Even some components of cell culture media such as riboflavin, tryptophan, tyrosine, pyridoxine and folic acid could generate ROS after light irradiation [63]. Therefore, to eliminate any interference and to quantify only the effects of the analyzed nanocomposites, the experimental system for the in vitro biocompatibility tests was kept in dark. The mechanism of ROS generation in visible light is well known, but hydroxyl radical and H_2_O_2_ production were recently reported in aqueous solution of photocatalysts even in dark [64,65].

The mechanism of ROS production in dark was described for the first time in 2015 by Lakshmi Prasanna and Vijayaraghavan [66], particularly for nano ZnO, involving superoxide species facilitated by surface defects. The mechanism of ROS generation in dark is almost similar with the light one. The only difference between dark and light ROS formation is that the singlet oxygen formation is not possible in dark because the hole required for its formation will not be produced in dark [66].

The cells own specific defense pathways that can keep the ROS levels corresponding to normal metabolic conditions and protect them against harmful oxidants. GSH represents a small molecule with a key role to maintain redox homeostasis; the reduced and oxidized form of glutathione complement other redox active compounds to regulate the cell redox state [67]. Our findings revealed that the photocatalyst samples used in this study induced oxidative stress that was revealed by an increased generation of ROS and a reduction of GSH levels. Exposure to all types of nanoparticles generated ROS in both types of human cells in a dose- and time-dependent manner. On the other hand, a major difference of toxicity between P25 Degussa NPs and the two TiO_2_/RGO photocatalysts was highlighted. P25 NPs induced in the both types of fibroblasts a much higher concentration of ROS compared to RGO-based nanocomposites suggesting that the newly-synthesized particles were more biocompatible with lung and dermal tissue. A possible explanation would be the ability of graphene oxide to adsorb proteins via electrostatic and hydrophobic interactions, which could attenuate its direct interactions with cell membranes, decreasing nonspecific binding with other functional biomolecules, leading to a lower cytotoxicity [68]. It is also obviously that the ROS generation is cell type dependent, because in MRC-5 cells regardless of nanoparticle type, it is higher than in CCD 1070Sk ones. As it was previously reported, the uptaken NPs could establish an interaction with NADPH (reduced nicotinamide-adenine dinucleotide phosphate) oxidase from cell membrane, resulting in superoxide anions formation in MRC-5 cells [69]. Dhaunsi et al. proved that *gp91-phox,* one of the two membrane components of NADPH oxidase found in skin fibroblasts, is not involved in the oxidative response [70]. In contrast, expression of NOX4, a homologue of *gp91-phox,* was observed in MRC-5 cells, and this could explain the difference in ROS generation between the two cell types.

However, the antioxidant defense system cannot completely prevent ROS formation and thus, damages on biomolecules can occur. Proteins and lipids, the most abundant molecules in living cells, represent the main targets of ROS attack. The elevation of AOPP level and protein carbonyls formation (Figure 15) in the skin and lung fibroblasts after 48 h exposure to photocatalysts, with the acute toxicity of P25 NPs in CCD 1070Sk cells and no major differences between the samples in MRC-5 cells, was positive correlated with the lipid peroxidation levels showed in Figure 14.

## 4. Materials and Methods

### 4.1. Synthesis of TiO_2_-Fe(1%)-N Samples

The synthesis of TiO_2_ NPs doped with 1% Fe and nitrogen was performed in hydrothermal conditions at 200 °C/2 h, using urea, in Teflon lined autoclave, following the general procedure described in our previous work [71]. The two samples discussed in this paper were synthesized by simultaneous co-precipitation (sample A) for uniform volume distribution of Ti and Fe ions and by sequential co-precipitation (sample B) with the aim to increase the probability for the iron ions to be localized at the particle surface.

For the synthesis of sample A, the calculated amounts of TiCl_3_ (15 wt. % TiCl_3_ in 10 wt. % HCl, Merck solution) and FeCl_3_·6H_2_O (p.a. Merck, Darmstadt, Germany) were dissolved and mixed simultaneously in water; NH_4_OH (25 wt. %, Merck, Darmstadt, Germany) was added up to pH ~ 9. Then Ti^3+^ was oxidized to Ti^4+^ under air bubbling to obtain Ti and Fe oxihydroxides. After washing and drying, the amorphous co-precipitate was placed in a 400 cm^−3^ Teflon lined autoclave with stirring. The hydrothermal treatment, in the presence of urea, was performed considering two temperature stages: 30 min at 105 °C followed by the final treatment at 200 °C for 120 min. The crystalline powder resulted after washing and drying was calcined at 400 °C for 120 min in air. 

For the synthesis of sample B, the precipitation process of Ti^3+^ and Fe^3+^ was sequential: in the first step, the Ti^3+^ ions were precipitated and oxidized to Ti^4+^ and then the Fe^3+^ ions were added to precipitate in a basic reaction medium (pH ~ 9).

### 4.2. Synthesis of TiO_2_/Reduced Graphene Oxide Nanocomposites

To obtain the TiO_2_-Fe(1%)-N/RGO composites a hydrothermal route was also used. A suitable amount of sample (A or B) was dispersed in distilled water using mechanical stirring and ultrasonication in the presence of poly diallyl dimethyl ammonium chloride (PDDA) (20 wt. %, molecular weight *M*w = 100,000–200,000, Sigma Aldrich, St. Louis, MO, USA). PDDA has a triplicate role as dispersing/reducing/stabilizer agent [72,73]. The excess amount of PDDA was eliminated and then the suitable quantity of GO (2 wt. % GO with respect to titanium oxide) was added. The resulted suspension was introduced in a 400 cm^3^ Teflon lined autoclave with stirring and kept at 150 °C for 120 min. The resulted precipitate was submitted to centrifugation and was dried at 80 °C for 24 h. Due to the strong reducing effect of PDDA, one obtains in fact RGO decorated with TiO_2_-Fe(1%)-N as evidenced by Raman spectroscopy (see Section 2.1.3 of this paper). Therefore, if GO is a monolayer material with high oxygen content (C/O ~ 3 or less), the RGO contains much less oxygen. For convenience, along this paper, the final two samples (TiO_2_-Fe(1%)-N decorated GO) obtained were denoted as sample A and sample B (the difference only consists in the precipitation route as mentioned above).

### 4.3. Characterization of Photocatalysts

The crystallization of photocatalysts, which were achieved using hydrothermal conditions followed by thermal annealing at 400 °C for 2 h, was revealed by XRD. A Bruker D8 Advance diffractometer (Bruker, Hamburg, Germany) was used with CuKα radiation (λ = 1.5406 Å). The iron in the TiO_2_ lattice was identified by ^57^Fe Mössbauer transmission spectra measured at room temperature using a WissEL-ICE Oxford Mössbauer cryomagnetic system (Wissenschaftliche Elektronik GmbH, Starnberg, Germany, and ICE Innovative cryogenic system, Oxford, UK). The Raman spectra of the TiO_2_/GO-based NPs were recorded in backscattering geometry using an FTRaman spectrophotometer, RFS100 model (Bruker, Hamburg, Germany).

TEM studies were performed on JEOL ARM 200F microscope using a voltage of acceleration equal to 200 kV and magnifications between 80,000× for Conventional TEM (CTEM) to 1,000,000× for High Resolution TEM (HRTEM).The photocatalytic properties of the samples A and B as thin films were investigated on a PCC-2 (ULVAC RIKO, Chigasaki, Kanagawa, Japan) photocatalytic checker. The thin films on Quartz buffer were obtained by dip coating technique with Xdip_SV1 apparatus (APEX INSTRUMENTS, Jadavpur Kolkata, West Bengal, India). The obtained films were dried in air for 24 h and then were cleaned to remove unlikely organic contaminants, by using a 30 W UV (385 nm) lamp for 2 h. The next step was to immerse the films in aqueous solution of methylene blue (1 mmol/L) for a suitable time; finally the samples were dried in dark at room temperature and then tested with PCC-2 checker. The machine generates pulsed light at 660 nm (which is also the main absorption of MB) and registers only the reflected pulses synchronized with the light emitting period in order to put on view the degradation of MB versus time, under UV or visible light. Absorbance ABS (*t*), which is a measure of photocatalytic degradation, is defined by the following formula:

ABS (*t*) = −ln [*I*(*t*)/*I*_0_], (1)

where *I*(*t*) is the intensity of reflected pulsed light (λ = 660 nm) at time “*t*” and *I_0_* is the light intensity at the beginning of the test.

As the pollutant MB placed on the photocatalytic film is degraded in time (under UV or visible irradiation) the reflected light intensity *I*(*t*) will be higher and higher. In agreement with the above formula, the ABS will be more and more negative. That means higher is the negative ABS value stronger is the photocatalytic activity of the tested photocatalytic material.

The photocatalytic activity was analyzed from the MB degradation curves (absorbance) obtained in both UV (368 nm) and visible (λ > 400 nm) spectral regions (~1 mW; at the irradiance of 2.5 W/m^2^).

### 4.4. Antimicrobial Activity Assays

The antimicrobial activity was tested on Gram-positive (*S. aureus* ATCC 6538) and Gram-negative (*P. aeruginosa* ATCC 27853 and *E. coli* ATCC 8739) bacterial as well as fungal (*C. albicans* 10231) reference strains. Microbial suspensions containing 1.5 × 10^8^ colony forming units (CFU) per mL (0.5 McFarland density) were obtained from 15 to 18 h bacterial cultures grown on solid media. The powders were solubilized in dimethyl sulfoxide (DMSO) and the starting concentration of the stock solution was 10 mg/mL. In order to determine the minimum inhibitory concentration (MIC) of the tested compounds, a quantitative assay was performed using the microdilution method in 96 multi-well plates. Specifically, serial two-fold dilutions of the solutions (ranging between 5 mg/mL and 0.15 mg/mL) were performed in 200 μL of sterile broth, and subsequently each well was inoculated with 50 μL of microbial suspension. Culture positive controls (wells containing medium seeded only with the microbial inoculum) were used. The influence of the DMSO solvent was also quantified in a series of wells containing only DMSO, diluted accordingly with the dilution scheme used for the tested complexes. Plates were incubated for 24 h at 37 °C in dark or under light exposure, and MIC values were calculated as the lowest concentration of the tested substance that inhibited the growth of the microbial overnight cultures, as compared to the positive control, revealed by a decreased value of absorbance at 600 nm [74,75,76].

To investigate the influence of the different concentrations on the ability of the tested microbial strains to establish colonization the inert substratum, the microplates previously used for the MIC assay were emptied and washed three times with PBS (phosphate buffered saline). Next, the biofilm that was formed on the plastic wall of the wells was fixed for 5 min using cold methanol, stained with violet crystal solution (15 min) and finally resuspended in a 33% acetic acid solution. The optical density of the colored solution which is an indicator of the cell density was read at 490 nm using an ELISA (enzyme linked immunosorbent assay) reader (Apollo LB 911). The lowest concentration of the tested substance that inhibited the biofilm development on the plate wells was considered to be the MBEC [77,78,79].

### 4.5. Cell Culture and Treatment

Normal human lung (MRC-5 cell line, ATCC Cat. No. CCL-171) and skin (CCD 1070Sk cell line, ATCC Cat. No. CRL-2091) fibroblasts were cultured in complete Eagle’s minimum essential medium (MEM; Gibco/Invitrogen, Carlsbad, CA, USA) with an addition of 10% fetal bovine serum (FBS; Gibco/Invitrogen, Carlsbad, CA, USA) at 37 °C, in a 5% CO_2_ humidified atmosphere. When the cells reached 70–80% confluence they were detached for sub-cultivation with a 0.25% (*w*/*v*) Trypsin and 0.53 mM EDTA solution (Sigma-Aldrich, St. Louis, MO, USA).

Two stock suspensions of graphene oxide decorated with two different kinds of TiO_2_ particles co-doped with 1% Fe-N atoms were prepared in phosphate buffered saline (PBS) and sterilized at 120 °C for 30 min. The cells were counted and seeded at a density of 3 × 10^4^ cells/cm^2^ into 24-well plates (for viability tests) or in 75 cm^2^ culture flasks (for oxidative stress analysis) and left to adhere overnight. Subsequently, cells were exposed to 100 and 200 μg/ml of TiO_2_-Fe(1%)-N-2% GO nanocomposites for 24 and 48 h. For each analysis controls (untreated cells) were performed. Following each treatment interval, the morphological changes of the fibroblasts were observed by phase contrast microscopy on an inverted Olympus IX71 (Olympus, Tokyo, Japan) microscope.

### 4.6. Cell Viability

The cell viability was determined by counting the cells using Trypan Blue staining. In this way, the cells were detached with a solution containing 0.25% (*w*/*v*) Trypsin and 0.53 mM EDTA (Sigma-Aldrich, St. Louis, MO, USA) and 15 μL of cell suspension were mixed with an equal volume of 0.4% (*w*/*v*) trypan blue solution prepared in 0.06% (*w*/*v*) dibasic potassium phosphate and 0.81% NaCl (Sigma-Aldrich, St. Louis, MO, USA). Blue stained cells (representing dead cells) and viable cells were counted using a dual-chamber hemocytometer and a light inverted microscope Nikon TS100 (Nikon, Tokyo, Japan). Cell viability was determined using the following formula:

% viable cells = [1.00 − (Number of dead cells/Number of total cells)] × 100,(2)

The amount of nitric oxide (NO) released in the culture medium as a marker of the inflammatory process was measured using the Griess reagent (a stoichiometric solution (*v*/*v*) of 0.1% naphthylethylendiamine dihydrochloride and 1% sulphanilamide in 5% H_3_PO_4_). Hence, culture supernatants were mixed with an equal volume of Griess reagent and stirred, whereupon the absorbance was read at 550 nm using a microplate reader (TECAN GENios, Grödic, Germany). NO concentration was determined by extrapolating results on a NaNO_2_ standard curve. 

Additionally, LIVE/DEAD Viability Assay Kit (L-3224, Invitrogen, Carlsbad, CA, USA) was also used to assess and image cell viability. After 24 and 48 h of treatment, cells were washed with PBS and stained with a mix solution composed of a calcein acetoxymethyl ester (calcein AM)and ethidium homodimer 1 (EthD-1) (2 μM:4 μM) for 10 min in dark, at room temperature. Following incubation, the cells were washed again with PBS and images of representative microscopic fields were visualized and captured using an inverted fluorescence microscope Olympus IX71 (Olympus, Tokyo, Japan).

### 4.7. F-actin Staining

Actin cytoskeleton morphology changes were visualized via fluorescence imaging of cells fixed with 4% paraformaldehyde for 20 min. Membrane permeability was achieved after 1 h incubation with 0.1% Triton X-100—2% bovine serum albumin (BSA). Filamentous actin (F-actin) was highlight using 20 μg/mL phalloidin conjugated with FITC (Sigma-Aldrich, St. Louis, MO, USA). Images were taken using an inverted fluorescence microscope Olympus IX71 (Olympus, Tokyo, Japan).

### 4.8. Cell Lysates Preparation

MRC-5 and CCD 1070Sk fibroblasts were harvested from culture flasks, rinsed with PBS and lysed by sonication (30 s × 3 cycles) on ice bath with an ultrasonic processor (Hielscher UP50H, Teltow, Germany). The cellular homogenate was centrifuged for 10 min at 3000× *g* and 4 °C. Forward, the supernatants (total proteic extracts) were retained for biochemical assays.

### 4.9. Protein Oxidation

The advanced oxidation protein products (AOPP) were quantified by the spectrophotometric method modified in our laboratory [80], using a chloramine-T standard curve. Briefly, 200 μL cell lysate were mixed with 10 μL of 1.16 M potassium iodide and incubated for 5 min at room temperature. Afterwards, 20 μL of glacial acetic acid were added in the mixture and shaken for 30 s. The absorbance of the samples was read at 340 nm using a microplate reader (TECAN GENios, Grödic, Germany). The AOPP concentration was expressed as nmol/mg protein and was represented relative to control.

The amount of protein carbonyl groups was determined according to the method optimized in our laboratory [80]. Thus, 500 μL of cell lysate diluted correspondingly were mixed with 500 μL of 10 mM 2,4-dinitrophenylhydrazine (DNPH) prepared in 2 M HCl and incubated at room temperature for 1 h. In the next step, 500 μL of ice-cold 20% TCA were added, keeping the mix on ice bath for 30 min. After a 3 min centrifugation at 13,000 rpm and room temperature, the supernatant was removed and the pellet was rinsed twice with 1 mL mixture consisting of ethanol: ethyl acetate (1:1) followed by centrifugation. After 10 min of incubation the resulting pellet was solubilized in 600 μL of 1 M NaOH and then incubated for 15 min at 37 °C. The absorbance of the samples was determined at 370 nm and the concentration of protein carbonyls was determined using the molar absorption coefficient of 22,000 M^−1^·cm^−1^. The final results were expressed in nmol/mg protein and were represented relative to control.

### 4.10. GSH Content

The proteins from cell lysates were precipitated with 5% sulfosalicylic acid (Sigma-Aldrich, St. Louis, MO, USA) and removed by 10 min centrifugation at 10,000 rpm and 4 °C. The GSH concentration was determined using a Glutathione Assay kit (Sigma-Aldrich, St. Louis, MO, USA) following the instructions provided by the manufacturer. Therefore, the samples were incubated with 5, 5′-dithiobis-2-nitrobenzoic acid (DTNB) at room temperature for 5 min to allow the reduction of DTNB into 5-thio-2-nitrobenzoic acid (TNB). The optical density was read at 405 nm using a microplate reader (TECAN GENios, Grödic, Germany). The GSH content was expressed as nmols/mg protein and the values were represented relative to control.

### 4.11. Lipid Peroxidation

Malondialdehyde (MDA) concentration was determined as a marker of lipid peroxidation using the fluorimetric method described previously by our group [81]. Thus, 200 µL of cell lysate, correspondingly diluted, were mixed with a volume of 700 µL of 0.1 N HCl and incubated at room temperature for 20 min. Further, 900 µL of 0.025 M thiobarbituric acid (TBA) were added in the mixture followed by 65 min incubation at 37 °C. Finally, the recorded relative fluorescence units (RFU) (excitation wavelength = 520 nm and emission wavelength = 549 nm) (FP-750 Spectrofluorometer, Jasco, Tokyo, Japan) were converted to nmols of malondialdehyde (MDA) using a standard curve of 1,1,3,3-tetramethoxypropane. Also, the MDA level was expressed as nmol of MDA/mg protein and all results were represented relative to control.

### 4.12. Intracellular ROS Level

The intracellular ROS level was assessed using a fluorescent compound 2′,7′-dichlorofluorescein diacetate (DCFH-DA, Sigma-Aldrich, St. Louis, MO, USA). The fibroblasts were washed with PBS and incubated with the dye for 30 min at 37 °C. Afterwards, the excess dye was cleared away and the cells were resuspended in PBS and detached by scraping. The fluorescence was quantified using a fluorimeter (FP-750 Spectrofluorometer, Jasco, Tokyo, Japan) (excitation wavelength = 488 nm and emission wavelength = 515 nm). All results were expressed relative to control after fluorescence intensity was reported to the number of viable cells of each sample.

### 4.13. Lysosomes Analysis

Lysosomes were stained following a previous described method [68], using LysoTracker Green DND-26 at a concentration of 100 nM (Molecular Probes, Invitrogen; excitation wavelength = 504 nm and emission wavelength = 511 nm) for 30 min at 37 °C and 5% CO_2_, and nuclei were counterstained with 2 µg/mL Hoechst 33342 (Molecular Probes, Invitrogen; excitation wavelength = 350 nm and emission wavelength = 461 nm) after 10 min incubation at room temperature. The images were captured using an inverted fluorescence microscope Olympus IX71 (Olympus, Tokyo, Japan).

### 4.14. Protein Concentration Assay

The protein concentration of the cell lysates was assessed using the Bradford Reagent (Sigma-Aldrich, St. Louis, MO, USA) and BSA as protein standard.

### 4.15. Statistical Analysis

Data were expressed as mean value ± SD of three independent experiments. Statistical differences between samples and control were evaluated by Student’s *t*-test using using the GraphPad Prism software (version 5; GraphPad Software, Inc., La Jolla, CA, USA), and a value of *p* < 0.05 was expressed as being statistically significant.

## 5. Conclusions

Novel TiO_2_/RGO-based NPs with enhanced photocatalytic properties were obtained by our group, and their great antimicrobial and low cytotoxicity have been reported within this study. By using a comprehensive approach, a detailed characterization of these photocatalysts showing the presence of unique iron and nitrogen co-doped anatase phase decorating RGO was reported, and the best photocatalytic activity for the sample with iron atoms localized at the sample surface. An in depth investigation of their effects triggered on cellular and molecular level has proved the low toxicity of nanocomposites compared to commercially P25 Degussa particles. These findings together with the great antibacterial potential confirm the high possibility of using these TiO_2_/RGO NPs to obtain more biocompatible and efficient materials for self-cleaning purposes, but also to open new exciting opportunities to improve human welfare.

## Figures and Tables

**Table 1:** Rietveld refinement parameters for TiO_2_/RGO samples.

Crystallographic Parameters	Samples		
	Sample A	Sample B	Bulk Anatase
a (Å)	3.7916(2)	3.7907(4)	3.7892
c (Å)	9.4869(3)	9.4872(1)	9.5370
c/a	2.5021	2.5208	2.5160
V (Å)^3^	136.40(6)	136.35(3)	136.93(3)
<Φ> (nm)	19.8	17.9	>>100 nm
ε (×10^4^)	13.1(7)	14.6(7)	-
Ti (4a)	(0,0,0)	(0,0,0)	(0,0,0)
O (8e)	(0,0,0.20989)	(0,0,0.20991)	(0,0,0.20790)
Phase assignment	Anatase	Anatase	Anatase

**Table 2:** Room temperature Mössbauer hyperfine parameters of the samples A and B.

Sample	IS ^1^ (mm/s)	ΔE_Q_^2^ (mm/s)	Γ ^3^ (mm/s)	Assignment
Sample A	0.392	0.530	0.45	Fe^3+^: TiO_2_ (anatase)
Sample B	0.454	0.599	0.49	Fe^3+^: TiO_2_ (anatase)
Errors	±0.002	±0.004	±0.05	

^1^ IS—isomer shift; ^2^ ΔE_Q_—quadrupole splitting; ^3^ Γ—Mössbauer line width.
